# Pulsed low-intensity ultrasound increases proliferation and extracelluar matrix production by human dermal fibroblasts in three-dimensional culture

**DOI:** 10.1177/2041731415615777

**Published:** 2015-11-19

**Authors:** Siti PM Bohari, Liam M Grover, David WL Hukins

**Affiliations:** 1 Faculty of Biosciences and Biomedical Engineering, Universiti Teknologi Malaysia, Johor Bahru, Malaysia; 2 School of Chemical Engineering, University of Birmingham, Birmingham, UK; 3 School of Mechanical Engineering, University of Birmingham, Birmingham, UK

**Keywords:** Alginate, collagen, glycosaminoglycan, human dermal fibroblast, proliferation, ultrasound, proliferation

## Abstract

This study evaluated the effect of pulsed low-intensity ultrasound on cell proliferation, collagen production and glycosaminoglycan deposition by human dermal fibroblasts encapsulated in alginate. Hoechst 33258 assay for cell number, hydroxyproline assay for collagen content, dimethylmethylene blue assay for glycosaminoglycan content and scanning electron microscopy were performed on the encapsulated cells treated with pulsed low-intensity ultrasound and a control group that remained untreated. Pulsed low-intensity ultrasound showed a significant effect on cell proliferation and collagen deposition but no consistent pattern for glycosaminoglycan content. Alcian blue staining showed that glycosaminoglycans were deposited around the cells in both treated and control groups. These results suggest that pulsed low-intensity ultrasound alone shows a positive effect on cell proliferation and collagen deposition even without growth factor supplements.

## Introduction

The healing of skin wounds involves several different tissues and cell lineages, including fibroblasts.^1^ It involves re-epithelialization, which includes the replication and movement of epidermal cells to make new tissue, followed by formation and contraction of granulation tissue consisting of small vessels, fibroblasts, myofibroblasts and inflammatory cells.^1–3^ Fibroblasts are responsible for the production and maintenance of the extracellular matrix (ECM) of connective tissues and appear to be activated by trauma.^4^

ECM is mainly responsible for the mechanical strength of tissues and also provides support for cells.^5,6^ It contains glycoproteins and glycosaminoglycans (GaGs) reinforced by collagen fibrils.^5–7^ Different tissues contain different GaGs and different collagens in varying proportions, to provide the required mechanical properties.^6,8^ Additionally, interactions between cells and the ECM are important for the function of a living tissue.^6,7,9^

In previous studies pulsed low-intensity ultrasound (PLIUS) was used to stimulate collagen production by 3T3 mouse fibroblasts^10^ and calf chondrocytes;^11^ this study investigates whether PLIUS can be used to stimulate human dermal fibroblast (HDF) activity. HDFs have been used to treat sun-damaged skin^12,13^ and for foetal skin repair.^14^ PLIUS stimulation has been shown to effect proliferation and matrix deposition of human chondrocytes^15,16^ and to increase GaG synthesis in a human nucleus pulposus cell line.^17^ It has also been suggested that PLIUS could improve wound healing in people suffering from diabetes mellitus, venous insufficiency and excessive pressure.^18^ However, the effect of PLIUS on HDF cells encapsulated in alginate has not been reported before. Encapsulation mimics the environment of a cell surrounded by ECM in a living organism.^19^ Alginate is a plant polysaccharide whose both chemical structure and physical properties resemble those of the GaGs in ECM; therefore, both alginate and collagen (the other main macromolecular component of ECM) have been used previously for encapsulation.^20–22^

## Materials and methods

### Materials

Alginate, Dulbecco’s modified Eagle medium (DMEM), 3-[4,5-dimethylthiazol-2-yl]-2,5-diphenyltetrazolium bromide (MTT) powder, Alcian blue dye, chloramine T, 1,9-dimethylmethylene blue dye (DMB), p-dimethylaminobenzaldehyde (p-DMBA) and a DNAQF DNA Quantification Kit were purchased from Sigma–Aldrich (Poole, Dorset, United Kingdom). Foetal calf serum was purchased from PAA Laboratories (Farnborough, Hampshire, United Kingdom). All other materials were obtained from Sigma–Aldrich.

### HDF cells

Fibroblasts were from a commercially available primary cell line (adult HDF; HPA Culture Collections, Porton Down, Salisbury, United Kingdom). Cells were maintained in DMEM supplemented with calf serum (10 v/v%), penicillin or streptomycin (1 v/v%), l-glutamine (2.4 v/v%) and 2-[4-(2-hydroxyethyl)piperazin-1-yl]ethanesulphonic acid (HEPES; 2.4 v/v%). Cells were stored in sterile conditions at 37°C and 5% CO_2_, and the growth medium was replaced every 3 days.

### Cell encapsulation

Cells were detached from the flask in which they were cultured with trypsin (0.05%) and EDTA (0.2 g/L) and resuspended in DMEM. Viable cell numbers were determined by staining with trypan blue and counting in a haemocytometer, to quantify the growth properties of the cells.^23^ After this, 2% sodium alginate solutions, made with double distilled water, were seeded with cells at a density of 2 × 10^6^ cells/mL.^24^ The cell suspension was transferred to the wells in 12-well plates and incubated in a bath of sterile CaCl_2_ solution for 2 h.^25^ The resulting discs of calcium alginate with encapsulated cells were washed three times with sterile phosphate-buffered saline solution (PBS) and transferred to six-well plate; each well contained supplemented DMEM (5 mL) which was refreshed every 3 days.^24^

### Cell encapsulation supplemented with growth factors

Cell were encapsulated and prepared in the same way as in the previous subsection, except that the cultures were supplemented with 50 µg/mL ascorbic acid^26^ and 2.5 ng/mL transforming growth factor beta-1(TGF-β1)^27^ to encourage ECM production.

### Ultrasound treatment

A Sonopuls 491 (Enraf-Nonius, Rotterdam, Amsterdam) ultrasound source was used. Its transducer was immersed in a water bath filled with deionized water maintained at 37°C^28^ and treated with a solution (Sigma Clean Water bath) to keep it free from bacterial and fungal growth. The deionized water was changed every week. The six-well plate containing constructs to be treated was placed on top of the transducer; the control group was maintained in the same conditions without being exposed to ultrasound. The ultrasound stimulation was performed 5 min every day for 10 days at the frequency of 1 MHz and an intensity of 0.2 W/cm^2^ with a 20% duty cycle (i.e. 20 pulses were emitted in 1 s, so that each pulse had a duration of 0.05 s).^10^ Treated and control samples were analysed, after 4, 6, 8 and 10 days, to determine cell numbers, collagen content and GaG content; at each time period, results for each analysis were obtained for three different samples.

### Cell content

Samples were freeze-dried for 2 days and then digested in papain solution (1 mL).^29^ Total cell numbers were determined by a Hoechst assay using a DNAQF DNA Quantification Kit.^30^ Hoechst 33258 dye (200 µL) was added to samples (10 µL) in a 96-well plate. Fluorescence (excited at a wavelength of 360 nm) was measured using a spectrophotometer (Promega GloMax; Promega, Southampton, United Kingdom) at a wavelength of 460 nm, at ambient temperature. The total cell numbers were obtained from a calibration curve constructed using HDF cells.

### Hydroxyproline assay

Hydroxyproline content provides a measure of collagen content and was determined using a method described previously^31,32^ but with some modification, as described below. In this method, 50 µL samples that had been digested with papain were hydrolysed in 6 M hydrochloric acid (HCl) at 110°C for 20 h and then kept in a vacuum dessicator overnight to allow evaporation of the remaining HCl. The samples were then reconstituted with 200 µL assay buffer (5 g/L citric acid (monohydrate), 12 g/L sodium acetate (trihydrate), 3.4 g/L sodium hydroxide and 1.2 mL/L glacial acetic acid in distilled water, pH 6.0).^31^ The reconstituted samples were then mixed with activated charcoal and left in ambient conditions for 30 min. They were then centrifuged at 14,000 r/min for 6 min. A total of 50 µL of the clear samples were then mixed with Chloramine T solution (50 µL, 62 mM) and then incubated at room temperature for 15 min to allow oxidation to occur. They were then mixed with 50 µL p-DMBA solution^32^ and incubated at 60°C for 30 min. The absorbance of the samples was measured using a microplate reader (Promega GloMax; Promega) at a wavelength of 550 nm. The hydroxyproline content of the samples were determined from a regression line of absorbance plotted against concentration (0–1 µg/mL) for trans-4-hydroxy-l-proline.

### GaG assay

The GaG content was quantified by a previously described method^10,33^ with a slight modification. Briefly, 40 µL of papain digested samples were added to 250 µL DMB dye at pH 1.5. This was done to minimize the reaction of the dye with the alginate.^34^ DMB dye can be influenced by binding of carboxyl groups of alginate to the DMB dye at pH 3.0.^33^ Absorbances were measured at a wavelength of 600 nm in the dark to maintain the stable condition of the dye^35^ using a spectrophotometer (Promega GloMax; Promega). The GaG content of the samples was determined from a standard solution of whale chondroitin 6-sulphate (0–100 µg/mL).

### Alcian blue staining

Alginate gels containing cells were stained using Alcian blue dye.^36,37^ The encapsulated cells were fixed with 10% formalin for 20 min and then washed with PBS. The gels were then stained with Alcian blue dye for 48 h (0.05% Alcian blue in 3% acetic acid, pH 1.5 and 0.3 M MgCl_2_).^36^ The alginate–cells were then washed sequentially with 3% acetic acid, 3% acetic acid and 25% ethanol, 3% acetic acid and 50% ethanol and 70% ethanol. The encapsulated cells were observed using a light microscope (AxioLab; Zeiss, Oberkochen, Germany).^36,37^

### Sample preparation for scanning electron microscopy

HDF cells encapsulated in an alginate disc were fixed with 2.5% glutaraldehyde in 0.1 M phosphate buffer for 1 h. After this, the samples were dehydrated in alcohol (50%, 70%, 90% and 100%) twice for 15 min for each alcohol change. Then, the samples were dried using critical point CO_2_, and the dried samples were mounted on a stub and coated with platinum. The samples were then viewed using a scanning electron microscope (SEM; Philips XL30 ESEM FEG; Philips, Eindhoven, the Netherlands).

### Statistical analysis

A Shapiro–Wilk test^38^ for normality was performed on all the data presented here. The data were then analysed using the independent t-test for normal data and the Mann–Whitney test for non-normal data.^38^ Differences were considered to be significant if the probability *p *< 0.05.

## Results

### Total cell number

**Figure 1.**
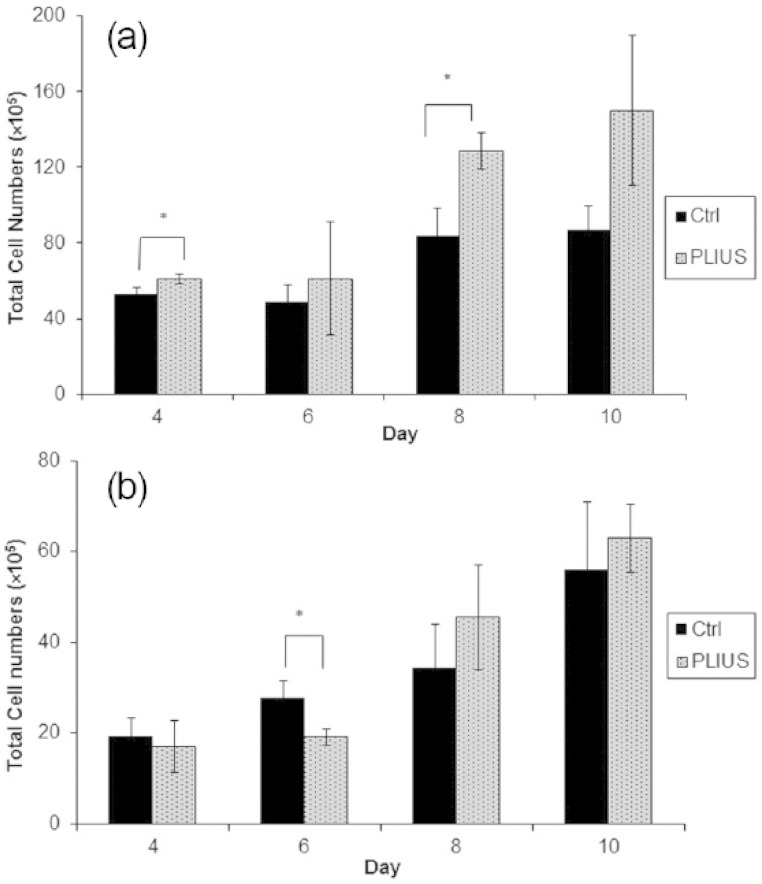
Cell proliferation for control (Ctrl) and treated (PLIUS) groups (a) not supplemented with growth factors and (b) supplemented with growth factors (ascorbic acid and TGF-β1). Results that are significantly different (*p *< 0.05) are marked with an asterisk.

Hoechst 33258 dye was used to quantify cell number since it is known to selectively stain double-stranded DNA for both live and dead cells as little as 5–10 ng in isolated nuclei, cells extracts and papain digestion samples;^39,40^ this is directly proportional to the total number of cells within a sample and is not influenced by the metabolic variations caused by encapsulation and PLIUS treatment. Using this assay, it was shown that samples treated with PLIUS contained higher cell numbers than the untreated samples in a period of 4–10 days (Figure 1(a)). However, this apparent difference was only statistically significant after 4 and 8 days. Figure 1(b) shows that when the samples are supplemented with TGF-β1, this apparent trend disappears; indeed the only statistically significant difference (after 6 days) shows fewer cells in the samples treated with PLIUS than in the control samples and suggests that the growth factor treatment has changed cell phenotype.

However, comparison of Figure 1(a) and (b) shows that PLIUS treatment alone is far more effective in increasing cell numbers than is supplementation with ascorbic acid and TGF-β1. After 10 days, the number of cells when growth factor was added was 63 × 10^5^ ± 7.5, which is comparable to the number (87 × 10^5^ ± 13) in control samples with no growth factors and no PLIUS treatment. However, the samples treated with PLIUS alone contain many more cells (150 × 10^5^ ± 40).

### Collagen production

**Figure 2.**
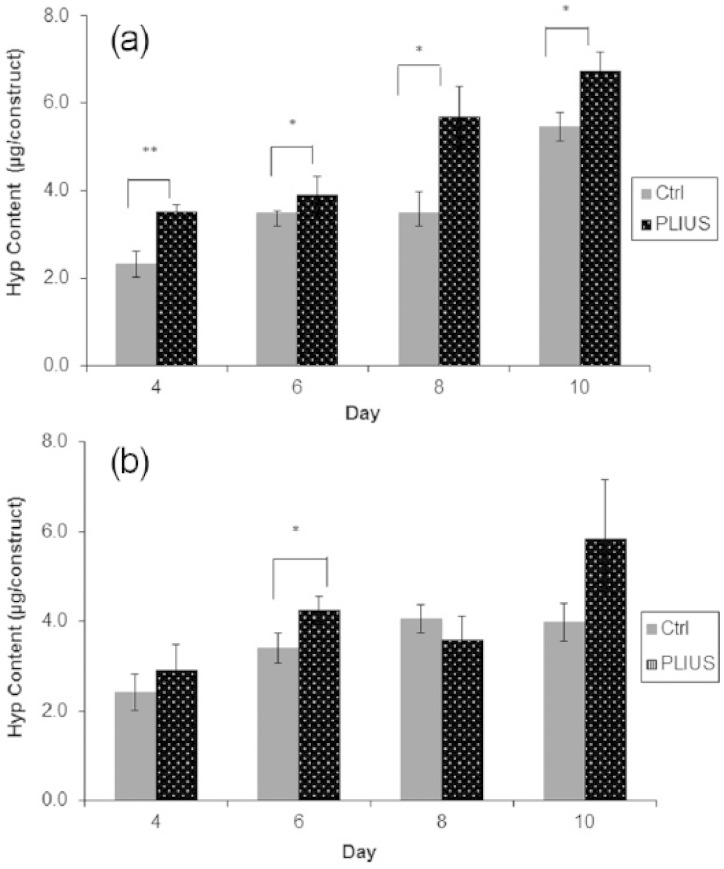
Hydroxyproline (Hyp) content for control (Ctrl) and treated (PLIUS) groups (a) not supplemented with growth factors and (b) supplemented with growth factors (ascorbic acid and TGF-β1). Results that are significantly different (*p *< 0.05) are marked with an asterisk; results that are significant at a higher level (*p *< 0.01) are marked with two asterisks.

Figure 2(a) shows a significant increase in hydroxyproline and, hence, collagen production throughout the 4- to 10-day period. However, Figure 2(b) shows that when the samples were supplemented with growth factors, no consistent trend is apparent, and PLIUS appears to significantly affect collagen production only at 6 days.

### GaG production

**Figure 3.**
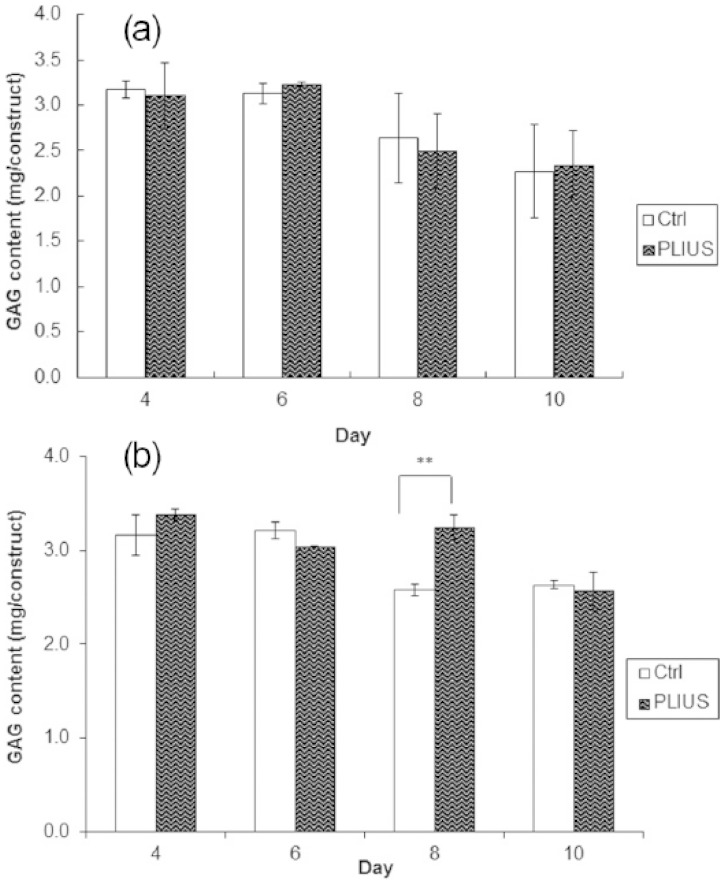
Glycosaminoglycan (GaG) content for control (Ctrl) and treated (PLIUS) groups (a) not supplemented with growth factors and (b) supplemented with growth factors (ascorbic acid and TGF-β1). Results that are significantly different (*p *< 0.05) are marked with an asterisk.

Figure 3(a) shows an apparent decrease in GaG content during the 4- to 10-day period with PLIUS having no statistically significant effect on the results. Similar results are shown in Figure 3(b) when the growth factors were added in the samples. Although PLIUS appears to significantly increase the GaG content at 8 days, there is no consistent pattern, and this single result is inconclusive.

**Figure 4.**
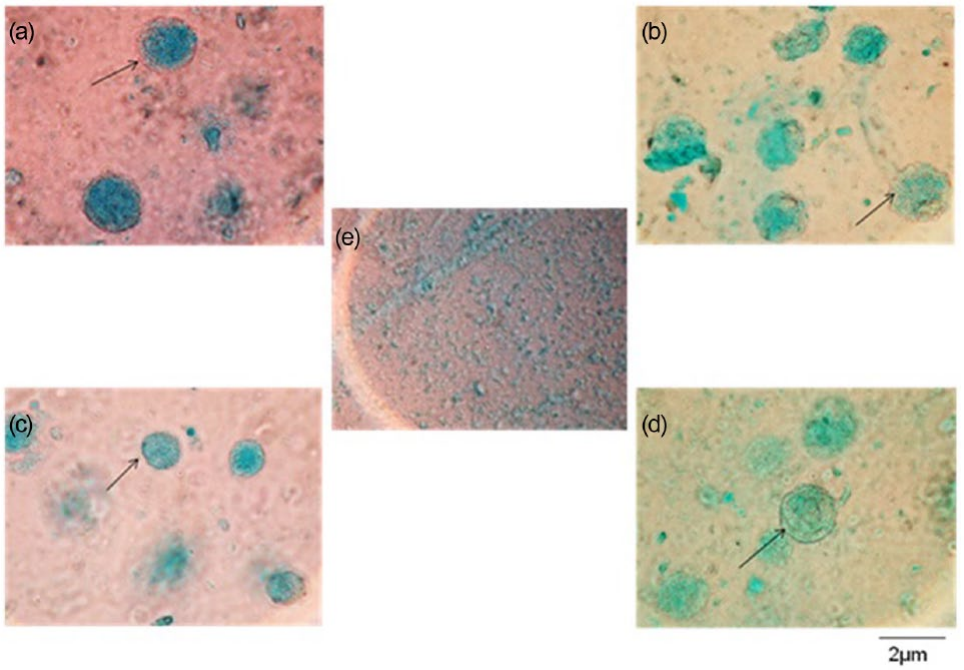
Alcian blue staining of human dermal fibroblasts encapsulated in alginate after 8 days in culture for (a) the control group in the absence of growth factors, (b) the treated group in the absence of growth factors, (c) the control group in the presence of growth factors, (d) the treated group in the presence of growth factors and (e) the alginate disc only. A blue dark region surrounding the cells shows the presence of GaG (arrow). All micrographs are at the same magnification.

GaG location in the encapsulated HDF cells was examined by Alcian blue staining. After 8 days as shown in Figure 4, the stained sample showed halos of Alcian blue around the cells indicating that they had begun to produce GaGs. Alcian blue staining revealed the formation of GaGs for the whole experimental period. Although the results were qualitative, they showed no difference in GaG production for control and treated groups.

### SEM result

**Figure 5.**
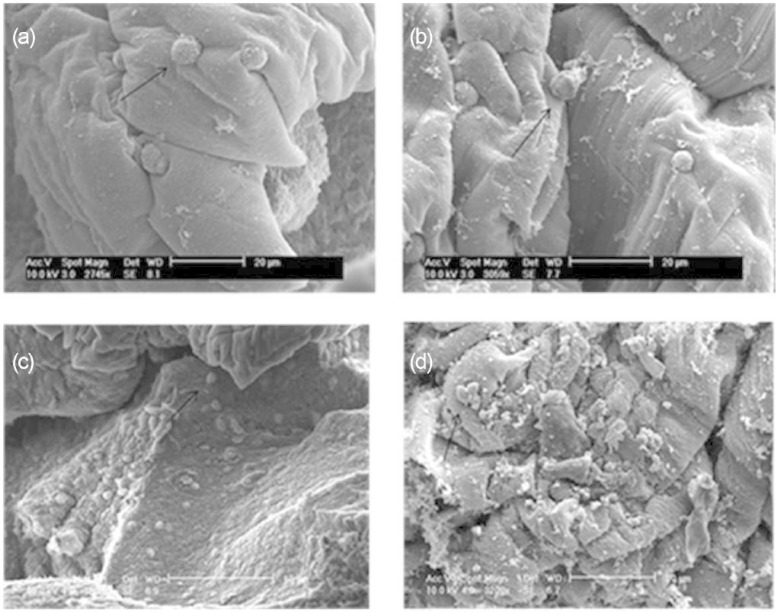
Scanning electron micrographs of a cross-section of alginate disc showing human dermal fibroblasts encapsulated in alginate after 10 days in culture for (a) the control group in the absence of growth factors, (b) the treated group in the absence of growth factors, (c) the control group in the presence of growth factors, (d) the treated group in the presence of growth factors and (e) the alginate disc only. A blue dark region surrounding the cells shows the presence of GaG (arrow). Examples of viable cells are indicated with an arrow.

As shown in Figure 5, the application of PLIUS to the cells did not appear to result in damage when compared to the control group. These results were comparable to those for the 3T3 cells.^10^ The cells did not spread, into a fibroblast-like shape, presumably because alginate does not contain moieties that allow for the attachment of cells. Interestingly, however, this does not appear to be a detrimental effect since the HDF cells maintained their viability and proliferated in culture (Figure 1(a) and (b)).

## Discussion

Many previous studies have shown a positive effect of therapeutic ultrasound, at different doses, on human fibroblasts.^18,28,41–45^ However, previous studies were all performed on monolayers of cells. In our previous report, we showed that therapeutic ultrasound increases collagen and GaG production for encapsulated mouse fibroblasts (3T3).^10^ The continuation study, described here, was intended to determine the effect of PLIUS on HDF cells maintained in three-dimensional (3D) culture.

The results for HDF cells show that PLIUS may lead to an increase in cell numbers but only in the absence of TGF-β1 and ascorbic acid. This finding is supported by previous studies that showed that therapeutic ultrasound increased the cell numbers for human fibroblasts.^28,43,44^ As discussed in the previous study,^10^ PLIUS did not stimulate the proliferation of 3T3 mouse fibroblasts. However, in the case of HDF cells, the results presented in this study show that PLIUS did stimulate cell proliferation but only in the absence of TGF-β1 and ascorbic acid. This is likely because although the PLIUS does provide a mechanical stimulus that can enhance proliferation, TGF-β1 promoted differentiation to a myofibroblastic phenotype. The myofibroblastic phenotype is characterized by the expression of α-smooth muscle actin (SMA) and an associated characteristic change in morphology to a stellate shape. Since the cells, however, were not attached to the alginate matrix, it was impossible to make a judgement on phenotype. Myofibroblasts are, however, responsible for the deposition of ECM proteins such as collagen I in wounds, although it should be noted that the deposition of matrix is usually at the expense of the rate of cell proliferation. Indeed, Doan et al.^28^ stated that if a cell number increase is observed with PLIUS treatment, this may actually reduce the extent to which the cells are able to deposit ECM. However, the data presented here were not totally in agreement with the results of Doan et al.^28^ since we demonstrated that an increase in cell number was associated with an increase in collagen deposition.

As shown in Figure 2(a), the hydroxyproline content and, hence, the collagen content increased for up to 10 days of treatment, but when the cells were supplemented with TGF-β1 and ascorbic acid (Figure 2(b)), there was no consistent effect. TGF-β1 functions by accelerating differentiation to matrix producing myofibroblast cells.^26,27,46–50^ Ascorbic acid has been shown to increase ECM synthesis in chondrocyte cells, human fibroblast and human aortic smooth muscle cells,^46,47,49^ while TGF β-1 was effective in upregulating the synthesis of collagen in human fibroblast cells, rabbit marrow mesenchymal stem cells and neonatal rat smooth muscle cells.^26,27,48,50^ However, the results presented here are inconsistent with these published observations. This may be due to the type of cells and scaffold being used and the culture conditions used to maintain the cells.^26,27,46–50^ Indeed, an article by Smith et al.^51^ demonstrated that when encapsulated in alginate, although cells can be shown to generate collagen type I, the influence of the highly charged alginate matrix inhibits collagen fibrillization within the gel structure, meaning that defective fibrils pass through the hydrogel matrix and into the surrounding matrix. This collagen is very difficult to quantify using chemical assays and has been observed to form on the surface of the culture media. Collagen that is accrued within these matrices seems to be deposited around the encapsulated cells, and where proliferation has occurred within pockets in the gel structure, increased quantities of collagen deposition have been observed. It is possible that the PLIUS stimulated proliferation within the gel enhanced collagen deposition in pockets within the gel, but the inhibited proliferation caused by myofibroblastic differentiation in the TGF-β1- and ascorbic acid–treated specimens meant that collagen production was maintained at a consistent level. Indeed, when one considers the quantity of collagen deposited within the matrix, it is directly related to cell number. This is clearly a very important consideration when designing a conditioning regime for generating collagenous 3D tissue constructs.

Treatment with PLIUS alone appeared to have no effect on GaG production (Figure 3(a)) and only shows a significant results (3.2 mg/construct) when used in conjunction with TGF-β1 and ascorbic acid (Figure 3(b)). While one might expect that TGF-β1 treatment might upregulate GaG production to a certain extent, the addition of ascorbic acid would be expected to have no effect since it is a cofactor in the intercellular processing of collagen prior to secretion. Furthermore, the pattern of the data here is also same as that of 3T3 mouse fibroblast cells in a previous study.^10^ This result could be explained by dye binding to the carboxyl groups in the alginate.^33^ Nevertheless, Alcian blue staining (Figure 4) showed that the HDF or alginate construct also produced the GaGs with or without the growth factor supplements.

SEM micrographs show that the cells encapsulated within the alginate disc did not adhere to the surrounding molecules (Figure 5). Previous studies also show that cells did not adhere to alginate^52,53^ unless it was modified by coupling with RGD peptides that mediate cell adhesion.^52,54^ Previous studies mentioned that the floating or aggregation of cells could be caused by the negatively charged guluronate and mannuronate residues in the alginate molecule.^22,53,55^ Alginate is highly porous material and is likely to entrap the cells within the matrix rather than the cells adhering to the alginate molecular chains.^22^ The lack of cell adhesion did not prevent the HDFs proliferating, even though the cells did not attach to the alginate (Figures 1(a), (b) and 5). This was supported by a previous study showing that MC3T3 cells also proliferate, and the differentiation is not modified when the cells were encapsulated within alginate beads.^56^ Moreover, hydroxyproline content also increased as a result of PLIUS treatment alone (Figure 2(a)) but not when it was combined with TGF-β1 and ascorbic acid (Figure 2(b)).This shows that the cells produce collagen when treated with PLIUS. When treated with both PLIUS and growth factors, the cells were involved less in cellular division (Figure 1(b)) rather than in collagen production (Figure 2(b)). Nonetheless, PLIUS alone performed better in enhancing cells proliferation (Figure 1(a)) and collagen production (Figure 2(a)).

It is noteworthy that under the conditions used in these experiments, PLIUS treatment did not show any deleterious effect on the cells. This is consistent with recent work in which PLIUS with a frequency of 1 MHz and intensities in the range of 0.75–1 mW/cm^2^ with 10%–20% duty cycles did not inhibit cell viability and proliferation.^28,43,44,57,58^ There is a possibility that the PLIUS conditions used in this experiment can be beneficial in wound healing;^18^ however, growth factors would be present in the healing wound. Given our unexpected finding that PLIUS is less effective in the presence of growth factors, this finding warrants further research.
